# *Schistosoma mansoni* infection risk for school-aged children clusters within households and is modified by distance to freshwater bodies

**DOI:** 10.1371/journal.pone.0258915

**Published:** 2021-11-04

**Authors:** Olimpia Lamberti, Narcis B. Kabatereine, Edridah M. Tukahebwa, Goylette F. Chami

**Affiliations:** 1 Big Data Institute, Clinical Trial Service Unit & Epidemiological Studies Unit, Nuffield Department of Population Health, University of Oxford, Oxford, United Kingdom; 2 Division of Vector Borne Diseases & Neglected Tropical Diseases, Ministry of Health, Kampala, Uganda; Dokkyo Medical University, JAPAN

## Abstract

**Background:**

The interaction of socio-demographic and ecological factors with *Schistosoma mansoni (S*. *mansoni)* infection risk by age and the household clustering of infections between individuals are poorly understood.

**Methods:**

This study examined 1,832 individuals aged 5–90 years across 916 households in Mayuge District, Uganda. *S*. *mansoni* infection status and intensity were measured using Kato-Katz microscopy. Socio-demographic and ecological factors were examined as predictors of infection status and intensity using logistic and negative binomial regression models, respectively, with standard errors clustered by household. A subgroup analysis of children was conducted to examine the correlation of infection status between children and their caretakers.

**Findings:**

Infection varied within age groups based on the distance to Lake Victoria. Children aged 9–17 years and young adults aged 18–29 years who lived ≤0.50km from Lake Victoria were more likely to be infected compared to individuals of the same age who lived further away from the lake. Infections clustered within households. Children whose caretakers were heavily infected were 2.67 times more likely to be infected.

**Conclusion:**

These findings demonstrate the focality of schistosome transmission and its dependence on socio-demographic, ecological and household factors. Future research should investigate the sampling of households within communities as a means of progressing towards precision mapping of *S*. *mansoni* infections.

## Background

Schistosomiasis is a neglected tropical disease (NTD) caused by blood flukes which are transmitted to humans indirectly through contaminated freshwater sources [1, 2]. Globally, over 250 million people are estimated to be infected with schistosomes, 750 million people are considered to be at risk of infection, and 1.9 million disability-adjusted life years are lost due to this chronic infection [3–5]. One of the most prevalent schistosome species infecting humans is *Schistosoma mansoni (S*. *mansoni)*, which causes intestinal schistosomiasis [1, 2, 5, 6]. People become infected when schistosome larvae penetrate the skin during water contact activities such as fishing, swimming, bathing, and wading [5, 7–9].

Mass drug administration (MDA) of praziquantel, whereby individuals are not diagnosed, is the most common treatment strategy for schistosomiasis and it is distributed at the school or community level [10–13]. Infection prevalence and intensity distributions against age are used to measure the burden of schistosome infection and to develop treatment guidelines for MDA [14]. It is well established that in endemic areas *S*. *mansoni* infection varies by age, whereby prevalence and intensity reach a peak in children, in particular those aged 10–14 years, and decline sharply in adults [15–21]. Based on this distribution, the WHO targets school-aged children (SAC) for MDA and national programmes focus treatment for individuals aged 5–14 years [14]. Yet, the age at which infection peaks is the result of complex interactions between the parasite life cycle, age-related exposure to infection, acquired immunity in the human host and, ecological and environmental conditions [15–21]. The peak of infection by age will vary across endemic communities. Shifts in the distribution of infection by age occur due to variations in parasite exposure and ongoing transmission related to socio-demographic and ecological factors [15–22].

The interaction of socio-demographic and ecological factors with age-related infection risk at the individual as opposed to the community level is poorly understood and quantitative empirical investigations are limited in endemic populations. It also is an open quesiton as to whether *S*. *mansoni* infections cluster within households. To examine the variation of age-related infection risk in priority treatment groups, we ask the following question. Is the risk of *S*. *mansoni* infection in SAC modified by socio-demographic, ecological, and household factors?

## Methods

### Participant sampling

This study is a secondary analysis of data collected in a community-wide survey of 30 villages on the shore of Lake Victoria in Mayuge District, Uganda [16]. For each village, 30 households were selected using systematic random sampling from a village register as described in Chami *et al*. [16]. One child (aged 5–17 years) and one adult/caretaker (aged 18+ years) per household were selected, resulting in 1,832 individuals from 916 households.

### Outcomes

Infection status of individuals was coded as a binary variable with a value of one if the individual was infected and zero if no eggs were observed. The *S*. *mansoni* infection intensity of individuals was defined as the number of eggs per gram (EPG) of stool. Each participant provided one stool sample which was analysed using Kato-Katz microscopy [23]. Two slides were prepared, and each read by a separate technician; egg counts were averaged and multiplied by 24 (slide size 41.7mg) to calculate EPG. Quality control readings were completed on 10% of the sample by a senior technician, and used to correct any slides where there was disagreement between the senior technician and the field technicians.

### Covariates

Socio-demographic covariates included the age and gender of individuals and the occupation of caretakers. Age ranged from 5–90 years old, excluding one child aged four who was included among individuals aged five years. Age was grouped into nine categories. Children (5–17 years) were divided into quartiles to the nearest age, and four groups were defined as follows: 5–8, 9–10, 11–12, and 13–17. Adults (18–90 years) were divided into quintiles resulting in the following groups: 18–29, 30–35, 36–40, 41–49, and 50+. The occupations of adults/caretakers were grouped into fishing (fishermen/fishmongers), rice-farmers, subsistence farmers and other occupations (including carpenters, businessmen and schoolteachers). These categories were selected to reflect the main activities in the study area [24].

Ecological covariates, which were coded at the village level, included the number of homes, number of roads, and binary variables for whether the village had a beach, landing site (small lake entry point only), or rice paddy, and access to at least one working public latrine or tap. In addition, the distances from the village centre to Lake Victoria and from the school to Lake Victoria were coded as binary indicators and equal to one if the distance was >0.50km and >1.0km, respectively. Descriptive statistics of variables used are reported in S1 Table in S1 File and the infection prevalence and intensity for different socio-demographic and ecological variables are provided in S2 Table in S1 File.

### Statistical analysis

Analyses were conducted using Stata v.16.1. Infection status and intensity were analysed using logistic and negative binomial regression models, respectively. All socio-demographic and ecological covariates were tested as potential predictors and effect modifiers (interactions between significant covariates) using likelihood ratio (LR) tests that controlled for the main exposure of age only (baseline models with age are shown in S3, S4 Tables in S1 File). Covariates and interactions that were significant (p-value≤0.05) were added to the final model. To understand whether age-related infection risk in children was associated with the characteristics of their caretakers, we conducted a subgroup analysis of children. WHO categories for light (1–99 EPG), moderate (100–399 EPG), and heavy (400+ EPG) *S*. *mansoni* infection intensity levels were constructed for the caretakers [16]. Socio-demographic characteristics of caretakers, village-level ecological variables, and the infection intensity level of caretakers were used to predict the infection status of children using logistic regression models. LR tests also were used to select socio-demographic and ecological predictors for the infection risk in children after adjusting for the age of children in the baseline model. All models were run with robust standard errors clustered by household [25]. This accounts for sampling by households and any potential correlation of outcomes within the sampling unit. The models were validated using 5-fold cross validation, and the area under the curve (AUC) and mean absolute errors (MAE) were calculated for the logistic regression and negative binomial regression, respectively.

### Ethics

The collection and use of this data were reviewed and approved by the Uganda National Council of Science and Technology (SS3082), Office of the president in Uganda (SS3082) and Cambridge University Human Biological Research Ethics Committee (HBREC2013.10) [16]. Parents or guardians provided consent on behalf of children.

## Results

### Infection variation by age and distance

**Fig 1:**
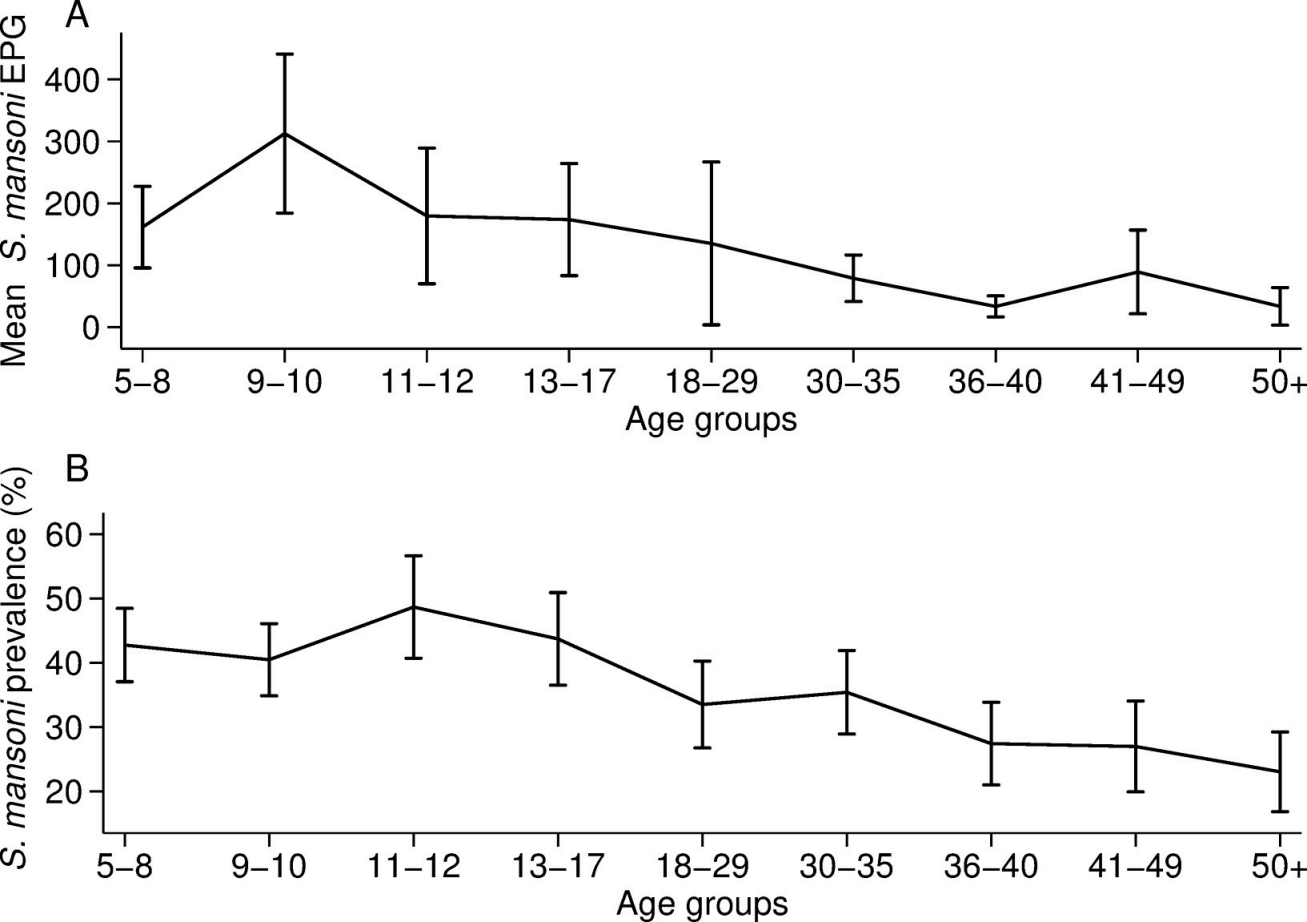
*S*. *mansoni* prevalence and infection intensity against age. A) Arithmetic mean of *S*. *mansoni* eggs per gram (EPG) for each age group with the corresponding 95% confidence intervals. B) *S*. *mansoni* prevalence for each age group with the corresponding 95% confidence intervals. In panel A, one child and one adult (aged 9 and 25 years) were outliers (>11,000 EPG) and were replaced with the next highest EPG in the age group. The number of individuals per age group are reported in S1 Table in S1 File.

The overall prevalence of *S*. *mansoni* was 36.4% (667/1832) and the mean intensity was 149.9 EPG (SD 676.8). Both infection prevalence and intensity were characterised by a peak in adolescence followed by a decrease in adults (Fig 1). The peak of infection intensity was in individuals aged 9–10 years-old, who had a mean intensity of 303.0 EPG (SD 1038.6) (Fig 1A). Infection prevalence peaked in late adolescence during 11–12 years of age, whereby 48.7% of individuals were infected (95% CI 40.7% - 56.7%) (Fig 1B).

**Fig 2:**
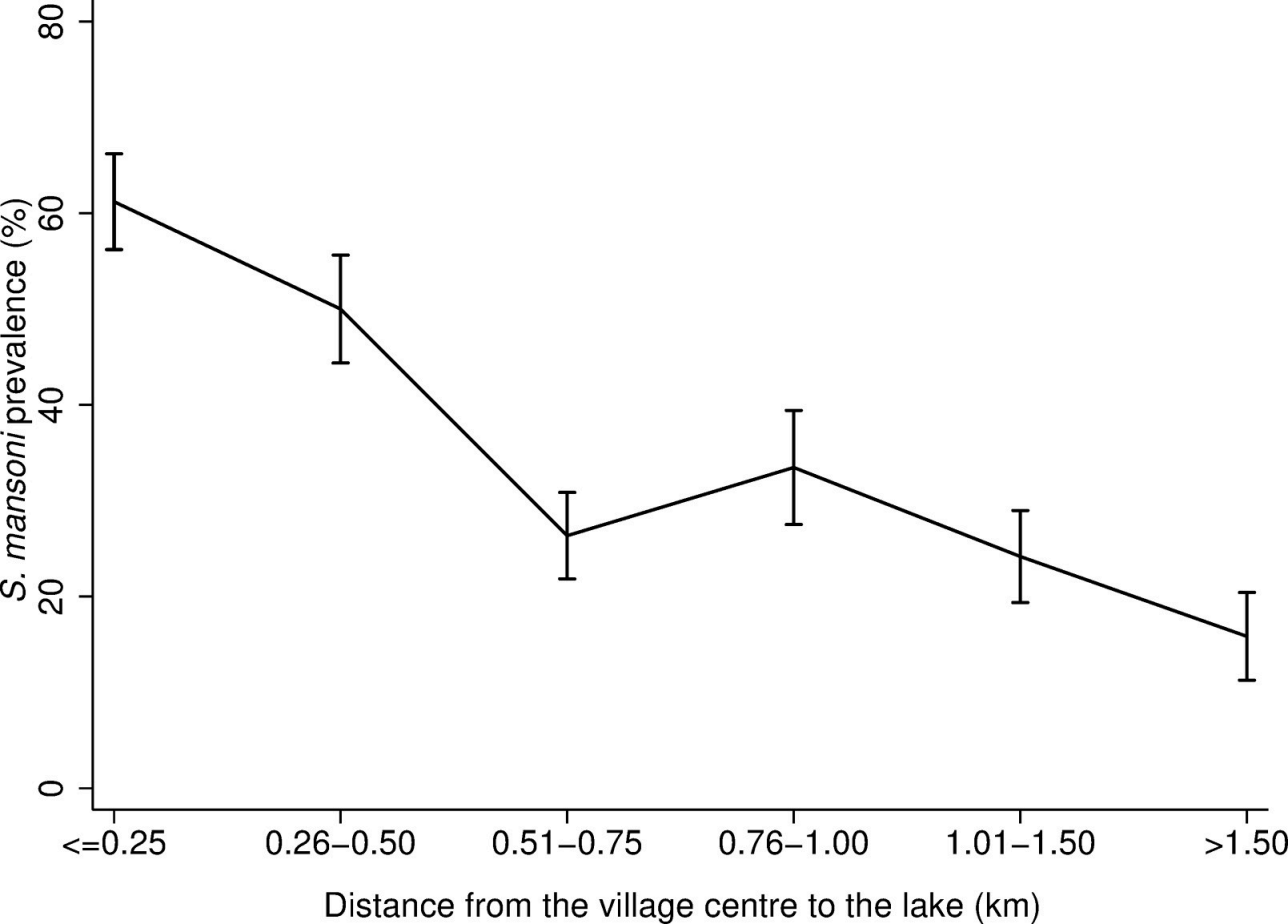
*S*. *mansoni* prevalence against distance from the village centre to Lake Victoria. *S*. *mansoni* prevalence for each category of distance from the village centre to the lake with the corresponding 95% confidence intervals. Here distance from the village centre to the lake has been divided in 0.25km bands in order to observe a more granular association. The number of observations per category of distance from the village centre to Lake Victoria are reported in S1 Table in S1 File. The maximum distance for any study village to Lake Victoria was 2.13km.

Over 70% (473/667) of infected individuals lived within 0.75km of Lake Victoria (Fig 2). For individuals living in villages within only 0.25km of Lake Victoria, 61.2% (224/366) of those individuals had a positive *S*. *mansoni* infection status. Among caretakers who lived within 0.25km from the lake, 17.5% (32/183) were involved in fishing and 53.0% (97/183) were subsistence farmers (S1 Fig in S1 File).

### Determinants of age-related infection

**Fig 3:**
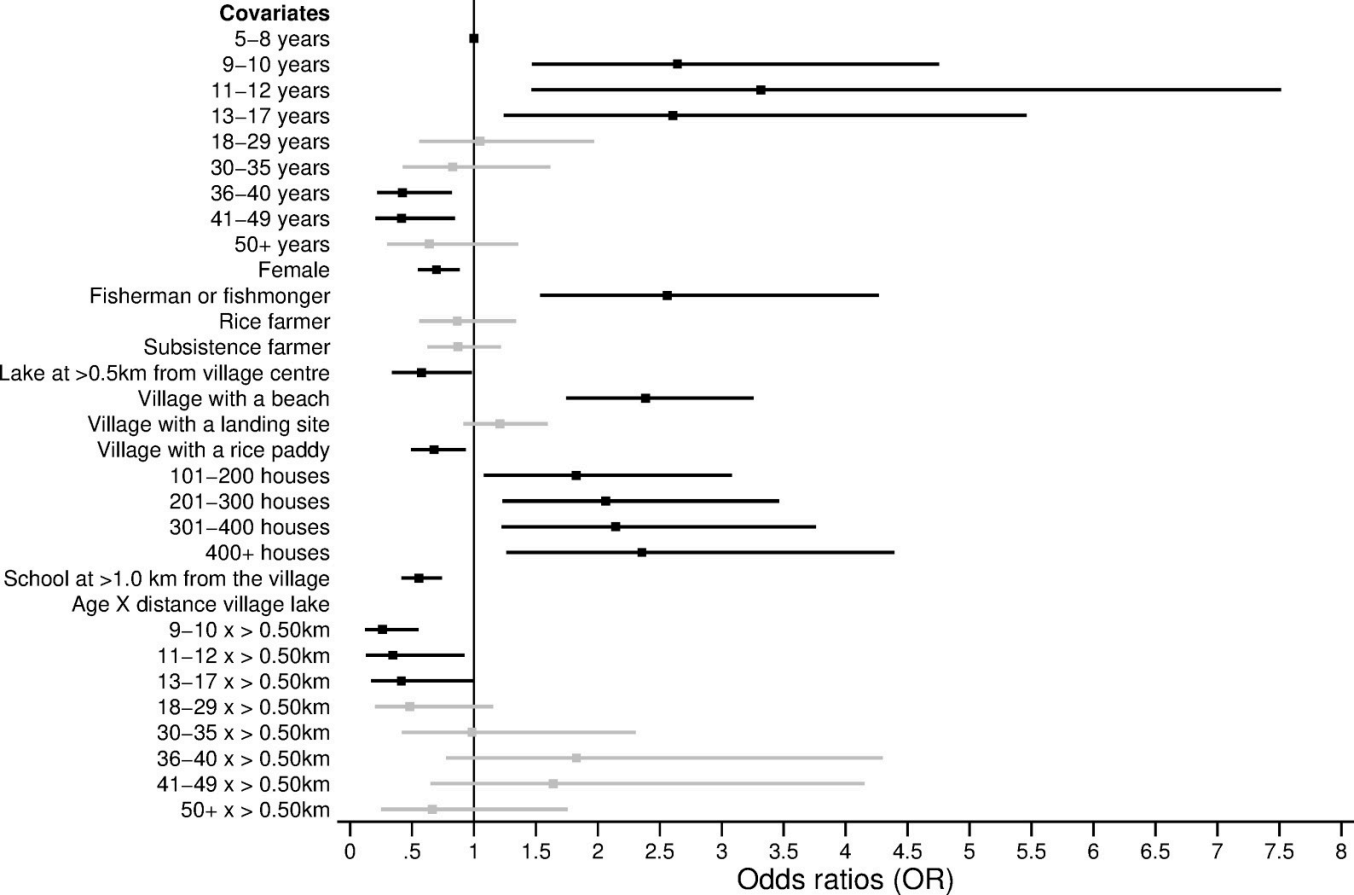
Predictors and modifiers of *S*. *mansoni* age-related infection status. Odds ratios (OR) are shown with the corresponding 95% confidence intervals (CI) from the logistic regression model presented in S5 Table in S1 File. Only covariates that were chosen through likelihood ratio tests are shown in the figure. The black squares (lines) represent the OR (95% CI) for the statistically significant covariates (p-value≤0.05). The grey squares (lines) represent the OR (95% CI) for the non-statistically significant (p-value>0.05) covariates.

**Fig 4:**
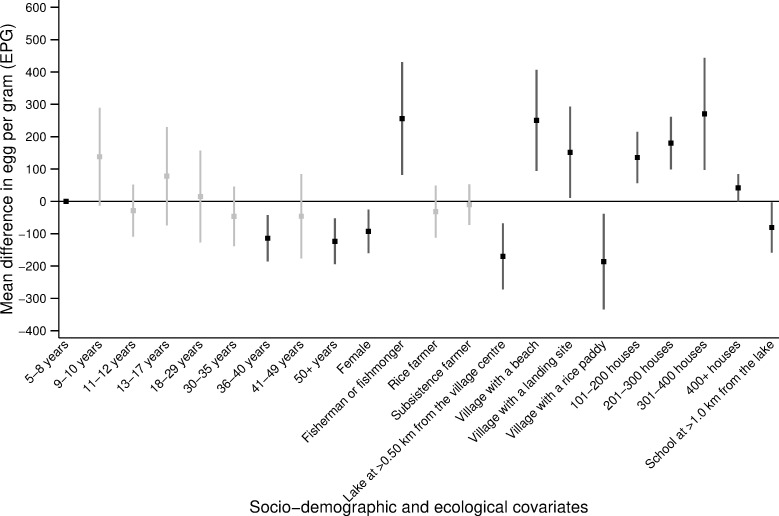
Predictors of the *S*. *mansoni* age-related infection intensity. Average change in eggs per gram (EPG), with the corresponding 95% confidence intervals (CI) from the negative binomial regression presented in S5 Table in S1 File. Only covariates that were chosen through likelihood ratio tests are shown in the figure. The black squares (lines) represent the average change in EPG (95% CI) for the statistically significant covariates (p-value≤0.05). The grey squares (lines) represent the average change in EPG (95% CI) for the non-statistically significant (p-value>0.05) covariates. Note: only four villages have 400+ houses.

Figs 3 and 4 present the predictors of *S*. *mansoni* infection status and intensity. The statistical models are reported in S5, S6 Tables in S1 File. Age, gender, and the occupation of the caretakers were significant predictors of infection status and intensity. Compared to children aged 5–8 years old, individuals aged 9–17 years were at higher risk of *S*. *mansoni* infection. In particular, children aged 11–12 years old were 3.32 times more likely to be infected with *S*. *mansoni* compared to children aged 5–8 years old (p-value = 0.004, Fig 3). Notably, children aged 5–8 years were not less likely (p-value>0.05) to have a lower infection intensity when compared to children aged 9–17 years (Fig 4). Across all age groups, females were 30% less likely to be infected compared to males (p-value = 0.003, Fig 3). After controlling for all significant ecological variables, including proximity to the lake, people belonging to a household where the caretaker was involved in fishing were 2.56 times more likely to be infected compared to individuals whose household head had other occupations (p-value<0.001, Fig 3). In terms of infection intensity, individuals in households with a fisherman or fishmonger had 250 more EPG compared to individuals in households with other occupations (p-value = 0.004, Fig 4).

**Fig 5:**
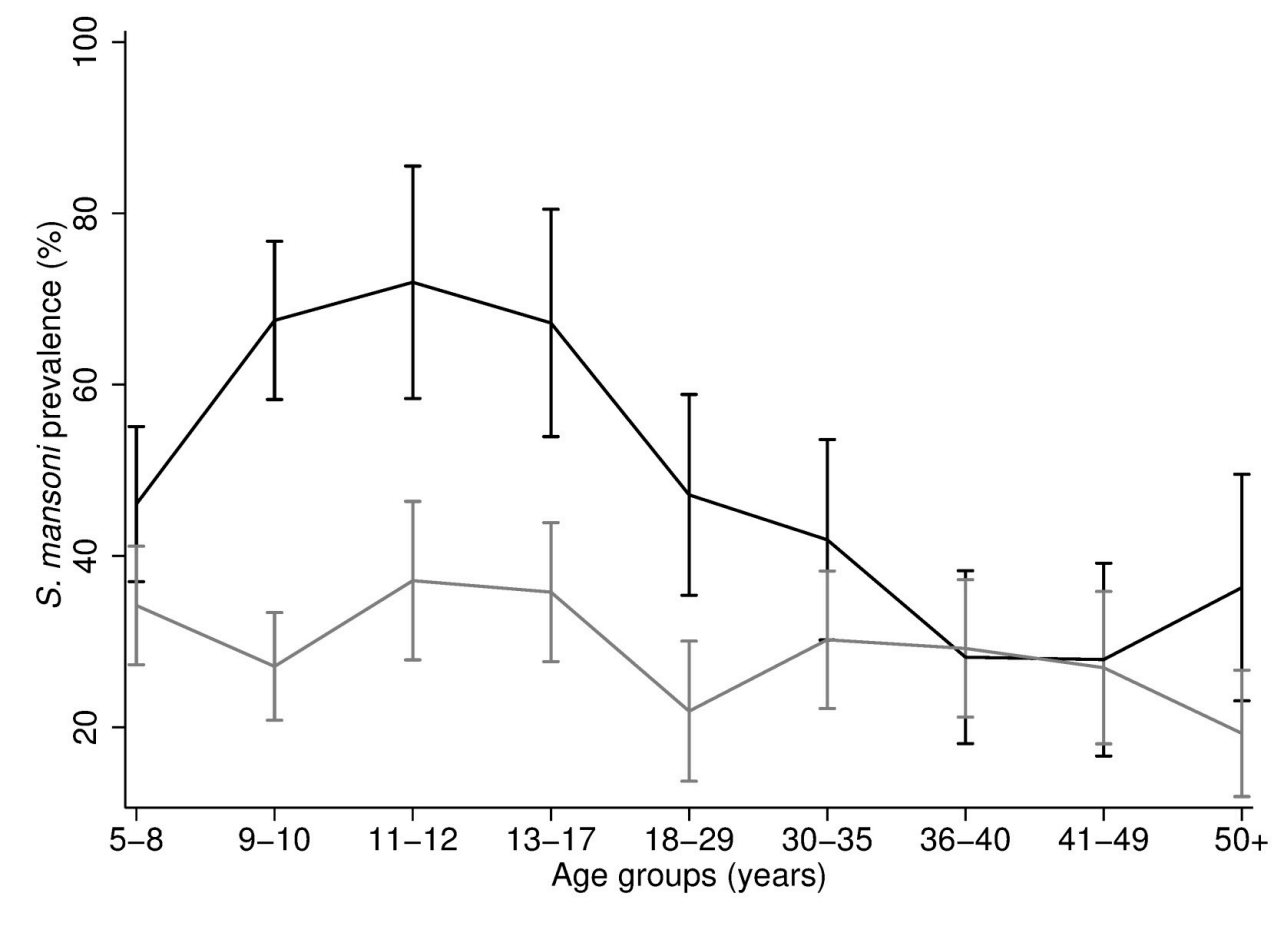
Interaction between age and distance from the village to Lake Victoria against infection status. The black line represents the infection prevalence across age groups for individuals located at a distance ≤0.50km from Lake Victoria. The grey line represents the *S*. *mansoni* infection prevalence for individuals located at a distance ≥0.50km from Lake Victoria. 95% confidence intervals are shown.

The number of houses in the village, access to a beach or rice paddy, and the distance from the village centre or primary school to Lake Victoria were significant ecological predictors of both infection status and intensity (p-value≤0.05). Individuals living in a village with access to a beach were 2.38 times more likely to be infected with *S*. *mansoni* compared to individuals in a village without a beach (p-value<0.001, Fig 3). These individuals also had 250.5 more EPG compared to individuals who had no direct access to the beach (p-value = 0.002, Fig 4). Similarly, individuals who lived at ≤0.50km from Lake Victoria were 42% (p-value = 0.04) more likely to infected with *S*. *mansoni* and had 170.2 EPG (p-value<0.001) higher intensity of infection compared to individuals living >0.50km from Lake Victoria (Figs 3 and 4). Proximity to the lake also modified the infection risk for children aged 9–17 years old and younger adults aged 18–29 years old (Fig 5). Individuals aged 9–29 years old who lived in a village located at a distance ≤0.50km from Lake Victoria had significantly higher odds of being infected compared to individuals in the same age group, who lived at a distance >0.50km from Lake Victoria (p-value≤0.05, Fig 5 and S5 Table in S1 File). More specifically, individuals who lived at ≤0.50km from Lake Victoria and were aged 9–29 years were, on average, 25–40% (p-value<0.001) more likely to be infected compared to individuals in the same age group who lived >0.50km from Lake Victoria (Fig 5 and S5 Table in S1 File).

### Household clustering of infections

**Fig 6:**
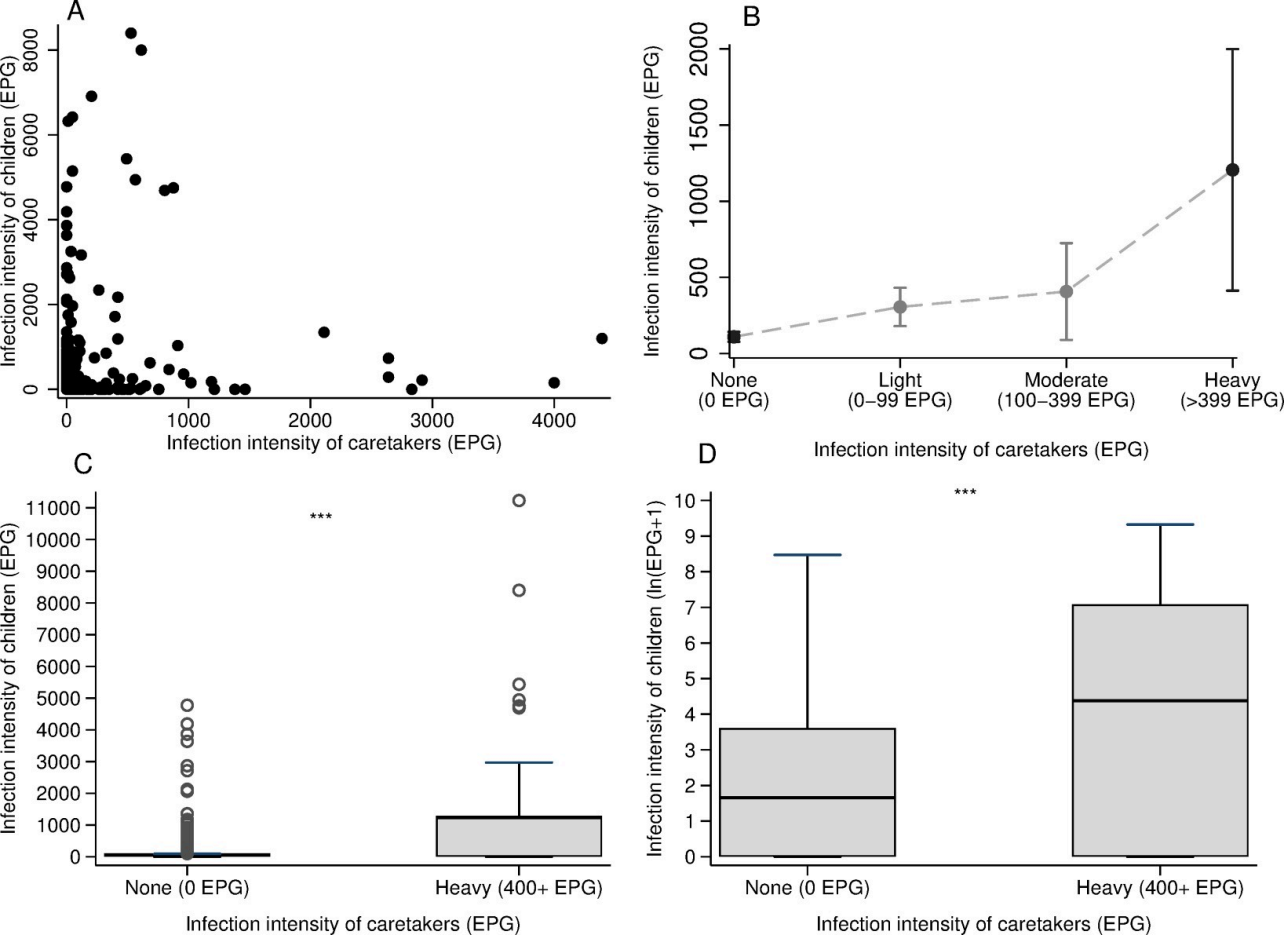
Mean *S*. *mansoni* infection intensity in children by the level of infection of their caretakers. **A)** Scatter plot of the distribution of infection intensities of children and caretakers. One child and one adult (outliers) with over 11,000 EPG were replaced to the closest highest number for EPG in this plot. **B)** Mean egg count of children with the corresponding 95% confidence intervals against the level of infection of caretakers. **C)** Box plot comparing the mean infection intensity for children whose caretakers were not infected (0 EPG) (Mean: 110.2 EPG; SD: 418.1) vs the mean infection intensity for children whose caretakers were highly infected (400+ EPG) (Mean: 1288.9 EPG; SD: 2526.6; two sample t-test = 9.9, p-value< 0.001***) **D)** Box plot comparing the log-transformed mean infection intensity for children whose caretakers were not infected (0 EPG)) (Mean: 1.7 ln(EPG+1); SD: 1.4) vs the mean infection intensity for children whose caretakers were highly infected (400+ EPG) (Mean: 4.4 ln(EPG+1); SD: 3.2; two sample t-test = 6.9, p-value<0.001***). The infection intensity of children was log-transformed as the raw distribution was highly skewed. In C and D, the boxes indicate the upper and lower interquartile range (IQR), the horizontal line in the box represents the mean, the whiskers represent -\+ 1.5*IQR and the hollow circles represent the data points with a value above 1.5*IQR.

**Fig 7:**
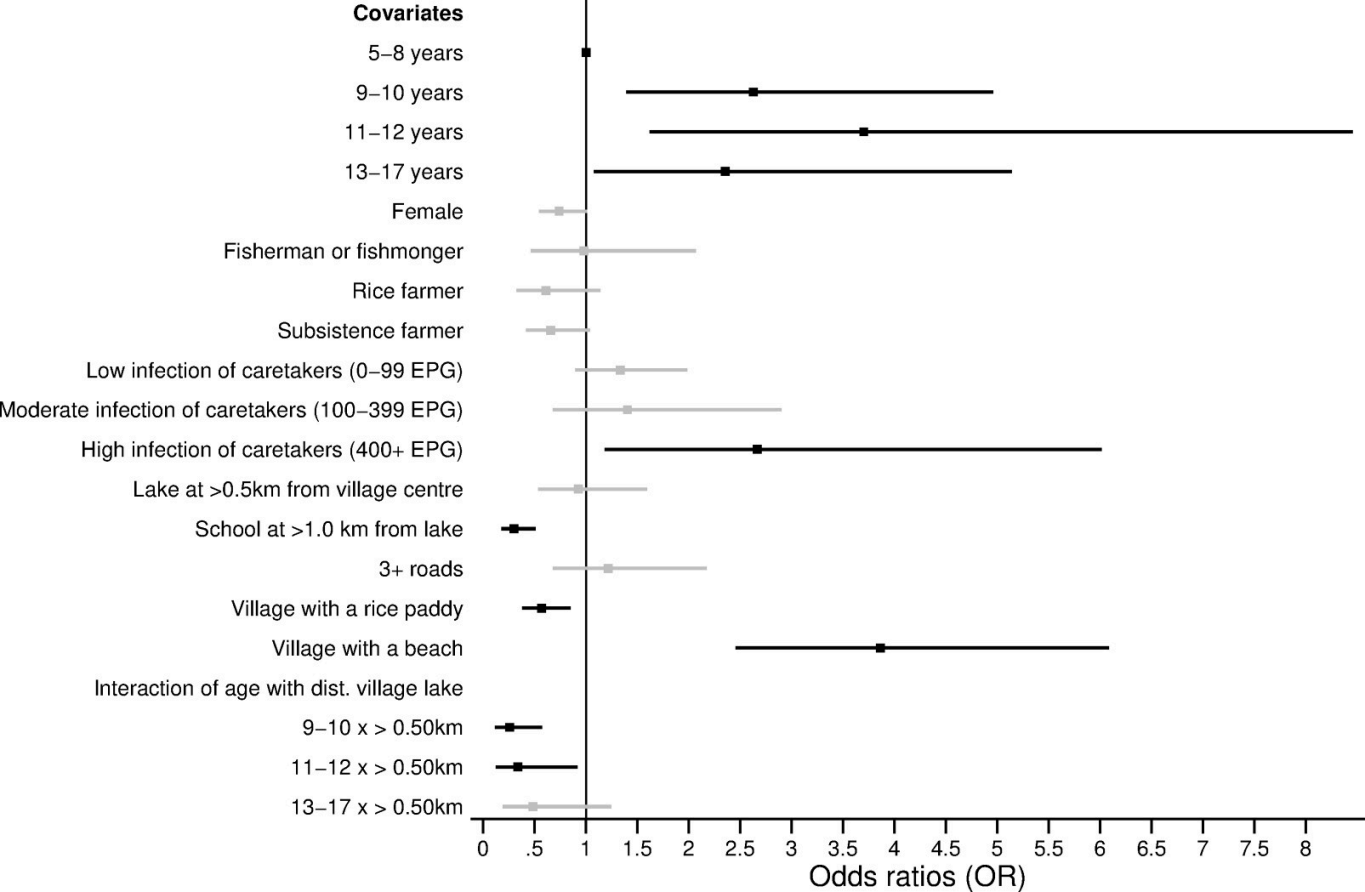
Predictors and modifiers of age-related infection status and intensity in children. Odds ratios (OR) with the corresponding 95% confidence intervals (CI) from the logistic regression model in S7 Table in S1 File are shown. Only covariates that were chosen through likelihood ratio tests are shown in the figure. The black squares (lines) represent the OR (95% CI) for the statistically significant covariates (p-value≤0.05). The grey squares (lines) represent the OR (95% CI) for the non-statistically significant (p-value>0.05) covariates.

There was evidence of clustering of *S*. *mansoni* infection at the household level (intraclass clustering coefficient = 0.17, p-value <0.001). The mean EPG in children was positively associated with the intensity of infection of their caretaker (Spearman’s rho = 0.26, p-value<0.001, Fig 6A, 6B). The mean intensity of infection was 177.6 EPG higher in children whose caretaker was heavily infected (400+ EPG) compared to children whose caretaker was not infected (t-statistic = -9.9, p-value<0.001, Fig 6C, 6D). Notably, children whose caretaker was heavily infected were 2.66 times (p-value = 0.02) more likely to have any infection compared to children whose caretaker was not infected (Fig 7; S7 Table in S1 File). This association was statistically significant even after controlling for the distance from the village centre to Lake Victoria and for the caretaker’s occupation. These results were observed despite the prevalence of infection being 13.2% higher in children compared to adults (χ^2^ = 35.0 p-value<0.001). The difference between children aged 5–8 years and those aged 9–17 years as well as the effects of ecological covariates that were found across all study participants remained robust in the subgroup analysis of children.

## Discussion

In Uganda, intestinal schistosomiasis is highly endemic in lakeside areas [1, 16–18]. While the transmission of *S*. *mansoni* infection is well-understood as resulting from contact with contaminated water, the exposure to infection is a complex process and an ongoing challenge for monitoring during MDA programmes [26]. The present study investigated the association of socio-demographic and ecological factors with the status and intensity of *S*. *mansoni* infection. A secondary analysis was conducted of 1,832 individuals aged 5–90 years across 30 villages along the shores of Lake Victoria in the Mayuge District, Uganda. We found that age, gender, occupation, and village-level ecological factors were significant predictors of the status and intensity of *S*. *mansoni* infection. Importantly, we showed that the infection status of children was associated with the level of infection of their caretakers.

The mean prevalence and intensity of *S*. *mansoni* infection peaked in two different age groups. Infection intensity peaked in individuals aged 9–10 years old while infection prevalence peaked in individuals aged 11–12 years. This differed from previous findings that described the peak in mean prevalence and intensity occurring at the same age [24, 27]. The distribution of infection against age here otherwise accords with well-established trends [1, 7, 18, 26] where the infection peaks in SAC and declines in adults. We found that the mean intensity of infection declined steadily with age, changing from 300+ EPG, in individuals aged 9–10 years, to 100–200 EPG in individuals aged 11–29 years, to <100 EPG in individuals aged 30+ years old. These observed patterns of infection might reflect the reduced susceptibility to reinfection for older children who have acquired immunity [26, 28]. Alternatively, these infection trends may be associated with the behavioural patterns of younger children who may have had prolonged contact with contaminated water, resulting in a higher intensity of infection [19, 29]. Understanding the patterns of infection across endemic areas is important for the design and implementation of treatment strategies. MDA programmes in Uganda are focusing on mass treatment of SAC with praziquantel [1, 11, 16]. However, we found that, despite a decade of treatment, intestinal schistosomiasis remains highly prevalent in SAC. Importantly, adults had a high prevalence of infection that ranged from 23.0% in individuals aged 50+ years to 33.5% in individuals aged 18–29 years. In 2002, the WHO recommended that adults in high-risk areas and women of child-bearing age be included in MDA programmes [30]. Yet, donations of praziquantel do not target adults, who are therefore not treated during most MDA campaigns [30, 31]. Transmission can continue from a few infected adults who contaminate the water [32]. This creates a reservoir of infection for children who may get infected when bathing and swimming [30]. Hence, adults must be included in the treatment and monitoring programmes for the control and elimination of schistosomiasis [30, 31].

Predictors of *S*. *mansoni* infection status included age, gender, occupation and village-level factors. Interestingly, we showed that compared to children aged 9–17 years, younger children aged 5–8 years, despite being less likely to be infected with *S*. *mansoni*, did not have a significantly different intensity of infection when actually infected. The higher likelihood of any infection in children aged 9–17 years-old may be due to several factors including the frequency and amount of water contact, whether it occurs in locations with infected snails, how much skin gets in contact with water, or the presence of age-related factors like acquired immunity and changes in skin texture [1, 18, 30, 33]. Despite children aged 5–8 years being less likely to be infected, the similar intensity of infection across children might be because younger children are more likely to be missed during MDA campaigns [34]. Children aged 5–8 years might be missed during MDA campaigns since they are in the transition period to begin primary school and may not yet be enrolled. This is particularly true in Uganda where, it is estimated that at the age of six, more than 18% of girls and 15% of boys are not enrolled in primary schools [35]. Any potential confusion in what constitutes school-aged versus pre-school aged might also result in missed treatment in younger children, given pre-school children (typically less than five years of age) are excluded from MDA. Further research is needed to discern why children aged 5–8 years have similar levels of infection intensity when compared to older children, including whether missed treatment is a possible reason for these similarities. In addition, we found that adults aged 36–49 years old were less likely to be infected with *S*. *mansoni* compared to children aged 5–8 years old. This may be due to acquired immunity in adults that make them less susceptible to infection [15, 36, 37]. Another possible explanation may be that adults aged 36–49 years old are less involved in occupations that involve prolonged and frequent contact with contaminated water, such as fishing. In our study population, most of the individuals involved in fishing were aged 18–35 years old. This may be partly why this age group did not show a statistically different risk of infection compared to the children aged 5–8 years.

The study design of paired observations within households enabled an analysis of how characteristics of caretakers influence *S*. *mansoni* infection risk in children [16]. Infection intensity was positively correlated within households. A child whose caretaker was heavily infected was 2.67 times more likely to be infected than a child who had an uninfected caretaker. This association may be due to the behaviours of caretakers that directly expose the children to *S*. *mansoni* infection and/or may reflect similarities in water contact patterns and activities among members of the same household [38]. For instance, children may follow their caretakers to the lake to assist them during domestic activities or to bathe themselves [38]. Interestingly, the occupation of adults did not determine this correlation of infection. Thus, it may be hypothesized that children did not assist their caretakers during their occupational activities. However, indirect transmission of infection might still occur from caretakers to their children due to shared water sources [30, 39, 40]. Children might bathe and swim in the same lake site as their infected caretakers and, if adults contaminate the water that children repeatedly visit then children will be at risk of infection [30, 41]. To test this hypothesis, future studies should measure the proximity from the household to transmission sites and track movements of children and their caretakers to approximate locations of water contact. Another possible explanation for the shared infection status within households is that the lower risk of infection within households may be associated with shared hygiene behaviours such as the use of soap whilst bathing in the freshwater body which confers some protection from *S*. *mansoni* infection by killing cercariae [7, 39, 40]. However, this study did not account for these factors and further analysis is needed to understand the effect of these factors on the likelihood of *S*. *mansoni* infection at the household level. Overall, results from the subgroup analysis reemphasise the need to expand treatment campaigns to adults [31].

Our analysis recorded higher prevalence and intensity of infection in males compared to females, similar to previous studies in Uganda [1, 16, 18]. Gender differences in infection have been found to be marked in places where socio-cultural factors result in one of the genders being more exposed to contaminated water [42]. The division of gender roles leads to women being involved in domestic and agricultural tasks and men to work in occupations involving frequent water contact such as fishing [18, 27, 42]. However, in our analysis, no significant interaction was found between gender and occupation, suggesting that the gender difference in infection prevalence and intensity did not depend on the occupation of individuals. These gender differences might therefore be explained by the timing, frequency, and duration of water contact activities whereby women have shorter and/or less frequent contact with infected water bodies compared to men [38]. In addition, we found that, across our study population, gender did not modify the age-related infection risk, suggesting that age and gender have two separate and independent effects on the infection status and intensity of an individual. This was further supported in the subgroup analysis where no difference in the likelihood of infection by gender was observed among children. This may reflect some gender similarities in exposure among individuals of similar ages who may go to the freshwater sources at similar times (for example before and/or after school for children) and may stay in the water for similar durations of time [38].

Proximity to the lake was found to be a significant risk factor for both *S*. *mansoni* infection status and intensity. The prevalence of schistosomiasis was higher for villages and schools closer to Lake Victoria when compared to villages and schools further away from the lake, which is consistent with previous studies in the same region [15, 43, 44]. One plausible explanation for this distance-infection relation is that individuals living closer to the lake have more frequent and prolonged contact with schistosome-infested waters [43]. For instance, in our study population, most adults involved in fishing lived within 0.50 km from Lake Victoria. Occupational exposures such as fishing may prolong the contact with contaminated waters, thus driving a higher prevalence of infection in the communities closer to Lake Victoria. In our analysis, proximity of the village centre to Lake Victoria was found to modify age-related infection risk in SAC and younger adults. Children aged 9–17 years old and young adults aged 18–29 years old who lived at >0.50km from the lake, had between 25% and 40% lower odds of being infected compared to children in the same age group who lived ≤0.50km from the lake. This entails a peak shift in the age-infection distribution to higher prevalence for children living closer to the lake compared to children of the same age living further from the lake. However, proximity from the village centre to the lake modified neither the effect of age against infection risk for the youngest children (aged 5–8 years) nor for adults aged 30+ years old. This may reflect differences in behaviours across age groups. It is plausible that older adults go to the lake frequently for occupational and domestic purposes despite the distance from the lake to their village. Differently, children aged 9–17 may spend more time at school and only children who go to school or live closer to the lake may go swimming after and/or during school hours. Interestingly, across our study population, proximity of the village centre to the lake did not modify the age-related infection intensity. Infection intensity is sometimes used as a measure of worm aggregation in an individual, which is the base for monitoring and evaluating in MDA programmes [36]. MDA campaigns targeting villages located closer to the lake may better reach SAC with higher rates of parasite exposure but, not necessarily, with a higher intensity of infection. There is a need to conduct more focal, precision mapping to reduce the size of MDA implementation units and to better locate high-risk populations [45–48]. One remaining challenge to consider is the mobility of populations. The districts along the shores of Lake Victoria are largely characterised by mobile, itinerant fishing communities [1]. This may undermine efforts to target individuals or households within those communities.

A limitation of our study is that an insensitive diagnostic procedure—Kato-Katz microscopy—was used to diagnose *S*. *mansoni* infections. This method was needed to classify infection intensities. Kato-Katz microscopy and point-of-care circulating cathodic antigen (POC-CCA) tests have been found to be in agreement for estimating community prevalence or infection status in high transmission areas for *S*. *mansoni* infections [49]. However, Kato-Katz microscopy is insensitive to very light egg-patent infections and ignores egg-negative infections. This may result in potentially misclassifying study participants who are infected as not infected. For very light egg-patent infections, we try to address this limitation partially with rigorous rereading of slides for quality control. For egg-negative infections, future research could resample participants over multiple days and compare these measurements to POC-CCA. This approach may also help address false positives from POC-CCA.

We demonstrated that infection risk clusters within a household, affecting not only children but also their caretakers. In addition, we showed that proximity to freshwater sources was a key predictor of infection risk and modified the effect of age against infection risk for SAC. Precision mapping of infection prevalence based not only on ecological foci, but also on household clustering is a promising way forward for MDA programmes to improve monitoring and evaluation guidelines at the community level.

## Supporting information

S1 File (DOCX)
