# Supplementary material for: Schistosoma mansoni infection risk for school-aged children clusters within households and is modified by distance to freshwater bodies
Source: PLoS One. 2021 Nov 4;16(11):e0258915. doi: 10.1371/journal.pone.0258915 (PMC8568121; doi:10.1371/journal.pone.0258915)
Supplement: S1 File — (DOCX) [file pone.0258915.s001.docx]

**File S1,** Supplementary information for *Schistosoma mansoni* infection risk for school-aged children clusters within households and is modified by distance to freshwater bodies

**Table of contents:**

[**Table S1:**Descriptive statistics of categorical and continuous variables used in the analysis 2](#_Toc74728185)

[**Table S2:** *S. mansoni* infection prevalence and intensity by socio-demographic and ecological variables 3](#_Toc74728186)

[**Table S3:** Logistic regression for *S. mansoni* infection risk adjusted for the age of individual. 4](#_Toc74728187)

[**Table S4:** Negative binomial regression for *S. mansoni* infection intensity adjusted for age only. 5](#_Toc74728188)

[T**able S5***:* Predictors and modifiers of *S. mansoni* age-related infection risk and intensity. 6](#_Toc74728189)

[**Table S6:** Association of socio-demographic and ecological predictors for *S. mansoni* infection intensity 7](#_Toc74728190)

[T**able S7:** Predictors and modifiers of age-related infection risk and intensity in children. 8](#_Toc74728191)

**Fig S1:** Individuals involved in fishing by distance from Lake Victoria……………………...........................9

| Table S1: Descriptive statistics of categorical and continuous variables used in the analysis | | | | | |
| --- | --- | --- | --- | --- | --- |
|  | **Categorical variables** | | **Continuous variables** | | |
| Variables | **Percent (%)** | **Frequency** | **Obs.** | **Mean** | **Std. Dev.** |
| Outcome variables |  |  |  |  |  |
| *S. mansoni* prevalence | 36.4 | 667/1832 |  |  |  |
| *S. mansoni* EPG |  |  | 1832 | 144.9 | 674.8 |
| Socio-demographic variables |  |  |  |  |  |
| Age groups |  |  |  |  |  |
| 5-8 | 15.8 | 290/1832 |  |  |  |
| 9-10 | 16.1 | 294/1832 |  |  |  |
| 11-12 | 8.3 | 152/1832 |  |  |  |
| 13-17 | 10.0 | 183/1832 |  |  |  |
| 18-29 | 10.3 | 188/1832 |  |  |  |
| 30-35 | 11.4 | 209/1832 |  |  |  |
| 36-40 | 10.2 | 186/1832 |  |  |  |
| 41-49 | 8.3 | 152/1832 |  |  |  |
| 50+ | 9.7 | 178/1832 |  |  |  |
| Gender |  |  |  |  |  |
| Female | 59.7 | 1094/1832 |  |  |  |
| Males | 40.3 | 738/1832 |  |  |  |
| Occupation of caretakers ^a^ |  |  |  |  |  |
| Fisherman or Fishmonger | 7.3 | 134/1832 |  |  |  |
| Rice farmer | 14.5 | 266/1832 |  |  |  |
| Subsistence farmer | 64.4 | 1180/1832 |  |  |  |
| Other occupations | 13.8 | 252/1832 |  |  |  |
| Ecological variables ^b^ |  |  |  |  |  |
| Village with a rice paddy | 76.7 | 1406/1832 |  |  |  |
| Village with a beach | 23.3 | 426/1832 |  |  |  |
| Village with a landing site only | 36.7 | 672/1832 |  |  |  |
| Village with at least one working public latrine | 13.3 | 244/1832 |  |  |  |
| Village with at least one working public tap | 59.8 | 1096/1832 |  |  |  |
| Distance from village  centre to the lake |  |  |  |  |  |
| <= 0.50 km | 36.6 | 670/1832 |  |  |  |
| >0.50 km | 63.4 | 1162/1832 |  |  |  |
| Distance from village  centre to the lake |  |  |  |  |  |
| <= 0.25 km | 20.0 | 366/1832 |  |  |  |
| 0.26-0.50 km | 16.6 | 304/1832 |  |  |  |
| 0.51-0.75 km | 20.1 | 368/1832 |  |  |  |
| 0.76- 1.0 km | 13.2 | 242/1832 |  |  |  |
| 1.01-1.50 km | 16.7 | 306/1832 |  |  |  |
| >1.50 km | 13.4 | 246/1832 |  |  |  |
| Distance from school to lake |  |  |  |  |  |
| <= 1.0km | 53.3 | 976/1832 |  |  |  |
| > 1.0 km | 46.7 | 856/1832 |  |  |  |
| Number of roads in the village |  |  |  |  |  |
| 1-2 | 56.6 | 1036/1832 |  |  |  |
| 3+ | 43.4 | 796/1832 |  |  |  |
| Number of houses in the village |  |  |  |  |  |
| <= 100 | 6.7 | 122/1832 |  |  |  |
| 101-200 | 36.6 | 670/1832 |  |  |  |
| 201-300 | 23.0 | 422/1832 |  |  |  |
| 300-400 | 20.2 | 370/1832 |  |  |  |
| 400+ | 13.5 | 248/1832 |  |  |  |

List of abbreviations: Obs. = Observations, Std. Dev. = Standard deviation, EPG = Eggs per gram

^a^ Household-level variable, representing the occupation of the household held

^b^ Village level variables

|  |  |  |  |  |  |  | | | |
| --- | --- | --- | --- | --- | --- | --- | --- | --- | --- |
| Table S2: S. mansoni infection prevalence and intensity by socio-demographic and ecological variables | | | | | | | | |  |
| Variables  N (%) or Mean (SD) | **With *S. mansoni* (667)** | **Without *S. mansoni*  (1,165)** | **Total  (1,832)** | **P- value** | ***S. mansoni EPG*** | | **VIF** |  |  |
| Socio-demographic variables |  |  |  |  |  | |  |  |  |
| Age groups |  |  |  | <0.001 |  | | 1.1 |  |  |
| 5-8 | 124 (42.8%) | 166 (57.2%) | 290 (15.8%) |  | 161.5 (572.4) | |  |  |  |
| 9-10 | 119 (40.5%) | 175 (59.5%) | 294 (16.1%) |  | 303.0(1038.6)* | |  |  |  |
| 11-12 | 74 (48.7%) | 78 (51.3%) | 152 (8.3%) |  | 179.6 (689.5) | |  |  |  |
| 13-17 | 80 (43.7%) | 103 (56.3%) | 183 (10.0%) |  | 175.5 (628.0) | |  |  |  |
| 18-29 | 63 (33.5%) | 125 (66.5%) | 188 (10.3%) |  | 87.0 (369.8)* | |  |  |  |
| 30-35 | 74 (35.4%) | 135 (64.6%) | 209 (11.4%) |  | 78.7 (277.1) | |  |  |  |
| 36-40 | 51 (27.4%) | 135 (72.6%) | 186 (10.2%) |  | 33.4 (118.0) | |  |  |  |
| 41-49 | 41 (27.0%) | 111 (73.0%) | 152 (8.3%) |  | 89.1 (425.5) | |  |  |  |
| 50+ | 41 (23.0%) | 137 (77.0%) | 178 (9.7%) |  | 33.4 (206.9) | |  |  |  |
| Gender |  |  |  | <0.001 |  | | 1.0 |  |  |
| Female | 349 (31.9%) | 745 (68.1%) | 1094 (59.7%) |  | 131.8 (731.8) | |  |  |  |
| Males | 318 (43.1%) | 420 (56.9%) | 738 (40.3%) |  | 164.3 (580.0) | |  |  |  |
| Occupation of caretakers |  |  |  | <0.001 |  | | 1.0 |  |  |
| Fisherman or Fishmonger | 93 (69.4%) | 41 (30.6%) | 134 (7.3%) |  | 468.7 (1151.9) | |  |  |  |
| Rice farmer | 74 (27.8%%) | 192 (72.2%) | 266 (14.5%) |  | 66.0 (372.5) | |  |  |  |
| Subsistence farmer | 392 (33.2%) | 788 (66.8%) | 1180(64.4%) |  | 99.0 (462.8) | |  |  |  |
| Other occupations | 108 (42.9%) | 144 (57.1%) | 252 (13.8%) |  | 179.5(589.5) | |  |  |  |
| Ecological variables |  |  |  |  |  | |  |  |  |
| Village with a rice paddy | 459 (32.7%) | 947 (67.4%) | 1,406 (76.7%) | <0.001 | 144.6 (566.2) | | 1.9 |  |  |
| Village with a beach | 238 (55.9%) | 188 (44.1%) | 426 (23.3%) | <0.001 | 299.7 (1008.0) | | 3.1 |  |  |
| Village with a landing site only | 221 (32.9%) | 451 (67.1%) | 672 (36.7%) | <0.001 | 108.9 (587.7) | | 1.4 |  |  |
| Village with at least one working  public latrine | 146 (59.8%) | 98 (49.2%) | 244 (13.3%) | <0.001 | 191.0 (677.7) | | 2.3 |  |  |
| Village with at least one working  public tap | 404 (36.9%) | 692 (63.1%) | 1,096 (59.8%) | 0.36 | 160.2 (659.0) | | 1.4 |  |  |
| Distance from village  centre to the lake | |  |  | <0.001 |  | | 1.7 |  |  |
| <= 0.50 km | 376(56.4%) | 294 (25.2%) | 670 (36.6%) |  | 262.2 (875.5) | |  |  |  |
| >0.50 km | 291 (43.6%) | 871 (74.8%) | 1,162 (63.4%) |  | 77.2 (513.7) | |  |  |  |
| Distance from village  centre to the lake |  |  |  | <0.001 |  | | 1.7 |  |  |
| <= 0.25 km | 224(61.2%) | 142 (38.8%) | 366 (20.0%) |  | 337.4 (979.9) | |  |  |  |
| 0.26-0.50 km | 152 (50.0%) | 152 (50.0%) | 304 (16.6%) |  | 158.6 (513.6) | |  |  |  |
| 0.51-0.75 km | 97(26.4%) | 271 (73.6%) | 368 (20.1%) |  | 67.4 (479.7) | |  |  |  |
| 0.76- 1.0 km | 81 (33.5%) | 161 (66.5%) | 242 (13.2%) |  | 161.6 (690.3) | |  |  |  |
| 1.01-1.50 km | 74 (24.2%) | 232 (75.8%) | 306 (16.7%) |  | 61.7 (368.4) | |  |  |  |
| >1.50 km | 39 (15.9%) | 207 (84.1%) | 246 (13.4%) |  | 14.9 (71.5) | |  |  |  |
| Distance from school to lake |  |  |  | <0.001 |  | | 2.7 |  |  |
| <= 1.0km | 459 (68.8%) | 517 (44.4%) | 976 (53.3%) |  | 209.8 (780.1) | |  |  |  |
| > 1.0 km | 208 (31.2%) | 648 (55.6%) | 856 (46.7%) |  | 70.9 (520.4) | |  |  |  |
| No. roads in the village |  |  |  | =0.001 |  | | 3.3 |  |  |
| 1-2 | 410 (39.6%) | 626 (60.4%) | 1,036 (56.6%) |  | 152.1 (660.0) | |  |  |  |
| 3+ | 257 (32.3%) | 539 (67.7%) | 796 (43.4%) |  | 135.4 (693.8) | |  |  |  |
| No. houses in the village |  |  |  | <0.001 |  | | 1.8 |  |  |
| <= 100 | 56 (45.9%) | 66 (54.1%) | 122 (6.7%) |  | 68.2 (178.6) | |  |  |  |
| 101-200 | 194 (29.0%) | 476 (71.0%) | 670 (36.6%) |  | 117.8 (652.7) | |  |  |  |
| 201-300 | 172 (40.8%) | 250 (59.2%) | 422 (23.0%) |  | 229.8 (854.3) | |  |  |  |
| 300-400 | 146 (36.5%) | 224 (60.5%) | 370 (20.2%) |  | 140.1 (656.1) | |  |  |  |
| 400+ | 99 (39.9%) | 149 (60.1%) | 248 (13.5%) |  | 118.3 (548.4) | |  |  |  |
| EPG = Eggs per gram. *Includes one outlier with EPG >11,000, which were replaced with the second highest EPG for that age group.  The p-value refers to the prevalence of *S. mansoni* infection and was calculated from the Pearson χ^2^ test. The Variance Inflation Factor (VIF) refers to the intensity of *S. mansoni* infection (EPG). | | | | | | | | |  |
| Occupations of caretakers are presented at the household level and represent the employment of the household caretaker | | | | | | | | |  |

**Table S3:** Logistic regression model for S. mansoni infection risk adjusted for the age of individuals only.

|  | Logistic regression model with infection status of individuals as an outcome | | | | |
| --- | --- | --- | --- | --- | --- |
| Variables | **Odds Ratios** | **Clustered**  **Std. Err.** | **P-value** | **95% CI** | |
| 5-8 years | 1.00 |  |  |  |  |
| 9-10 years | 0.91 | 0.15 | 0.58 | 0.65 | 1.27 |
| 11-12 years | 1.27 | 0.26 | 0.24 | 0.86 | 1.89 |
| 13-17 years | 1.03 | 0.20 | 0.84 | 0.72 | 1.51 |
| 18-29 years | 0.67 | 0.13 | 0.04 | 0.46 | 0.99 |
| 30-35 years | 0.73 | 0.14 | 0.10 | 0.51 | 1.01 |
| 36-40 years | 0.51 | 0.10 | 0.001 | 0.34 | 0.75 |
| 41-49 years | 0.50 | 0.11 | 0.001 | 0.32 | 0.76 |
| 50+ years | 0. | 0.09 | <0.001 | 0.26 | 0.61 |
| List of abbreviations: Clustered Std. Err. = Clustered Standard Error, CI= confidence intervals  Number of observations: 1,832 | | | | | |

**Table S4:** Negative binomial regression model for S. mansoni infection intensity adjusted for the age of individuals only.

|  | Negative binomial regression model with infection intensity of individuals as an outcome | | | | |
| --- | --- | --- | --- | --- | --- |
| Variables | **Average Marginal Effect** | **Clustered**  **Std. Err.** | **P-value** | **95%CI** | |
| 5-8 years | - |  |  |  |  |
| 9-10 years | 140.2 | 68.7 | 0.04 | 5.60 | 274.7 |
| 11-12 years | 18.1 | 65.1 | 0.78 | -109.4 | 145.7 |
| 13-17 years | 12.2 | 57.0 | 0.83 | -99.6 | 124.1 |
| 18-29 years | -47.4 | 57.9 | 0.41 | -160.9 | 66.0 |
| 30-35 years | -82.7 | 39.0 | 0.03 | -159.1 | -6.2 |
| 36-40 years | -128.0 | 34.6 | <0.001 | -195.8 | -60.3 |
| 41-49 years | -72.4 | 46.1 | 0.12 | -162.8 | 18.0 |
| 50+ years | -129.0 | 37.0 | 0.001 | -200.5 | -55.5 |
| List of abbreviations: Std. Err. = Standard Error, CI= confidence intervals  Number of observations: 1,832 | | | | | |
| The marginal effects were derived from the negative binomial regression model and represent the average change in eggs per gram (EPG) for individuals in different age groups compared to children aged 5-8 years old | | | | | |

| Table S5*:* Predictors and modifiers of *S. mansoni* age-related infection risk and intensity. | | | | | | | | | | |
| --- | --- | --- | --- | --- | --- | --- | --- | --- | --- | --- |
|  | **Model 1- Logistic Regression model**  **Outcome: Infection status** | | | | | **Model 2- Negative binomial regression model**  **Outcome: Infection intensity** | | | | |
| Covariates | **Odd Ratios** | **Clustered Std. Err.** | **P-value** | **95% CI** | | **Average Marginal Effect** | **Clustered**  **Std. Err.** | **P-value** | **95% CI** | |
| 5-8 years | 1.00 |  |  |  |  |  |  |  |  |  |
| 9-10 years | 2.64 | 0.79 | <0.001 | 1.47 | 4.75 | 138.08 | 77.21 | 0.074 | -13.25 | 289.41 |
| 11-12 years | 3.32 | 1.38 | <0.001 | 1.46 | 7.51 | -28.50 | 41.05 | 0.488 | -108.97 | 51.96 |
| 13-17 years | 2.60 | 0.98 | 0.01 | 1.24 | 5.46 | 78.00 | 77.53 | 0.314 | -73.95 | 229.96 |
| 18-29 years | 1.05 | 0.34 | 0.88 | 0.56 | 1.97 | 15.12 | 72.39 | 0.835 | -126.76 | 156.99 |
| 30-35 years | 0.83 | 0.28 | 0.58 | 0.43 | 1.61 | -46.52 | 47.07 | 0.323 | -138.78 | 45.74 |
| 36-40 years | 0.42 | 0.14 | 0.01 | 0.22 | 0.82 | -114.02 | 36.52 | 0.002 | -185.59 | -42.44 |
| 41-49 years | 0.42 | 0.15 | 0.02 | 0.21 | 0.85 | -45.96 | 66.51 | 0.490 | -176.32 | 84.39 |
| 50+ years | 0.64 | 0.25 | 0.24 | 0.30 | 1.36 | -123.71 | 36.08 | 0.001 | -194.42 | -52.99 |
| Female | 0.70 | 0.08 | <0.001 | 0.55 | 0.88 | -92.60 | 34.41 | 0.007 | -160.03 | -25.16 |
| Fishermen or fishmonger | 2.56 | 0.67 | <0.001 | 1.54 | 4.27 | 255.91 | 88.95 | 0.004 | 81.56 | 430.25 |
| Rice farmer | 0.87 | 0.19 | 0.51 | 0.56 | 1.34 | -31.78 | 40.91 | 0.437 | -111.96 | 48.41 |
| Subsistence farmer | 0.87 | 0.15 | 0.42 | 0.63 | 1.22 | -9.98 | 31.91 | 0.754 | -72.53 | 52.57 |
| Village with a rice paddy | 0.68 | 0.12 | 0.017 | 0.49 | 0.93 | -186.54 | 75.40 | 0.013 | -334.33 | -38.75 |
| Village with a landing site | 1.21 | 0.17 | 0.18 | 0.92 | 1.59 | 151.59 | 72.00 | 0.035 | 10.49 | 292.70 |
| Village with a beach | 2.38 | 0.38 | <0.001 | 1.75 | 3.25 | 250.52 | 79.63 | 0.002 | 94.44 | 406.60 |
| 101-200 houses in the village | 1.83 | 0.49 | 0.024 | 1.08 | 3.08 | 135.72 | 40.56 | 0.001 | 56.22 | 215.22 |
| 201-300 houses in the village | 2.06 | 0.54 | 0.006 | 1.23 | 3.46 | 180.17 | 41.53 | <0.001 | 98.76 | 261.57 |
| 301-400 houses in the village | 2.14 | 0.61 | 0.008 | 1.22 | 3.76 | 270.83 | 88.36 | 0.002 | 97.65 | 444.01 |
| 400+ houses in the village | 2.36 | 0.75 | 0.007 | 1.26 | 4.39 | 41.84 | 21.78 | 0.055 | -0.85 | 84.53 |
| School at > 1.0km from the village centre | 0.56 | 0.08 | <0.001 | 0.42 | 0.74 | -80.88 | 39.67 | 0.041 | -158.62 | -3.13 |
| Lake at >0.50km from the village centre | 0.58 | 0.16 | 0.043 | 0.34 | 0.98 | -170.17 | 52.04 | 0.001 | -272.16 | -68.18 |
| Age X distance village lake |  |  |  |  |  |  |  |  |  |  |
| 9-10 x >0.50 km | 0.26 | 0.10 | <0.001 | 0.12 | 0.55 |  |  |  |  |  |
| 11-12 x >0.50 km | 0.35 | 0.17 | 0.03 | 0.13 | 0.92 |  |  |  |  |  |
| 13-17 x >0.50 km | 0.41 | 0.19 | 0.05 | 0.17 | 1.01 |  |  |  |  |  |
| 18-29 x >0.50 km | 0.48 | 0.21 | 0.10 | 0.20 | 1.15 |  |  |  |  |  |
| 30-35 x >0.50 km | 0.98 | 0.43 | 0.97 | 0.42 | 2.30 |  |  |  |  |  |
| 36-4 x >0.50 km | 1.83 | 0.80 | 0.17 | 0.78 | 4.30 |  |  |  |  |  |
| 41-49 x >0.50 km | 1.64 | 0.78 | 0.30 | 0.65 | 4.15 |  |  |  |  |  |
| 50+ x >0.50 km | 0.66 | 0.33 | 0.41 | 0.25 | 1.75 |  |  |  |  |  |
| List of abbreviations: Std. Err. = Standard Error, CI= confidence intervals. Number of observations: 1,832  Standard errors were clustered at the household level. There were 916 clusters. For Model 2, the standard errors for the marginal effects are calculated from clustered standard errors in the negative binomial regression model.  The area under the curve (AUC) value for 5-fold cross validation of the logistic regression model was 0.74. This value considered the clustering of SE and were therefore similar to the 5-fold AUC values when observations were grouped at the household level (AUC=0.75)  The mean absolute error (MAE) for 5-fold cross validation of the negative binomial regression was 141.6. This value considered the clustering of SE and were therefore similar to the 5-fold MAE values when observations were grouped at the household level (MAE=141.5) | | | | | | | | | | |
| Model 1 reports the odds ratios of the logistic regression model for *S. mansoni* infection risk. Model 2 presents the marginal effects for *S. mansoni* infection intensity. Marginal effects measure the change in EPG for a one-unit change in the specific socio-demographic and ecological variables. Only variables that were chosen through likelihood ratio tests are shown in each model. | | | | | | | | | | |

**Table S6:** Association between socio-demographic and ecological predictors for *S. mansoni* infection intensity

|  | Negative binomial regression model – Outcome: *S. mansoni* infection intensity | | | | |
| --- | --- | --- | --- | --- | --- |
| Variables | **Negative binomial**  **regression coefficient** | **Clustered  Std. Err.** | **P-value** | **95%CI** | |
| 5-8 years (base category) | . | . | . | . | . |
| 9-10 years | 0.59 | 0.29 | 0.04 | 0.02 | 1.17 |
| 11-12 years | -0.18 | 0.26 | 0.49 | -0.70 | 0.33 |
| 13-17 years | 0.38 | 0.34 | 0.26 | -0.28 | 1.04 |
| 18-29 years | 0.08 | 0.39 | 0.85 | -0.68 | 0.85 |
| 30-35 years | -0.32 | 0.33 | 0.34 | -0.97 | 0.33 |
| 36-40 years | -1.11 | 0.33 | 0.001 | -1.76 | -0.45 |
| 41-49 years | -0.31 | 0.49 | 0.63 | -1.29 | 0.65 |
| 50+ years | -1.29 | 0.33 | <0.001 | -1.93 | -0.65 |
| Female | -0.57 | 0.18 | 0.001 | -0.92 | -0.22 |
| Fishermen or fishmonger | 1.00 | 0.27 | <0.001 | 0.49 | 1.53 |
| Rice farmer | -0.24 | 0.32 | 0.43 | -0.87 | 0.38 |
| Subsistence farmer | -0.07 | 0.22 | 0.75 | -0.50 | 0.36 |
| Village with a rice paddy | -0.96 | 0.28 | 0.001 | -1.50 | -0.41 |
| Village with a landing site | 0.77 | 0.27 | 0.005 | 0.23 | 1.29 |
| Village with a beach | 1.67 | 0.23 | <0.001 | 0.72 | 1.61 |
| 101-200 houses in the village | 1.63 | 0.38 | <0.001 | 0.89 | 2.37 |
| 201-300 houses in the village | 1.87 | 0.32 | <0.001 | 1.24 | 2.49 |
| 301-400 houses in the village | 2.22 | 0.38 | <0.001 | 1.47 | 2.97 |
| 400+ houses in the village | 0.82 | 0.40 | 0.04 | 0.04 | 1.60 |
| School at > 1.0km from the village centre | -0.57 | 0.40 | 0.07 | -1.20 | 0.05 |
| Lake at >0.50km from the village centre | -1.14 | 0.32 | <0.001 | -1.65 | -0.63 |
| List of abbreviations: Std. Err = standard error, CI= confidence intervals  Number of observations 1,832  Standard errors were clustered at the household level. There were 916 clusters. | | | | | |

| Table S7: Predictors and modifiers of age-related infection risk and intensity in children. | | | | | | | | | | | | | | | | |
| --- | --- | --- | --- | --- | --- | --- | --- | --- | --- | --- | --- | --- | --- | --- | --- | --- |
|  | **Logistic regression models – Outcome: infection status in children** | | | | | | | | | | | | | | | |
|  | **Model 1** | | | | | **Model 2** | | | | | **Model 3** | | | | | |
| Variables | **Odds**  **Ratios** | **Std. Err.** | **P-value** | **95%CI** | | **Odds**  **Ratios** | **Std. Err.** | **P-value** | **95%CI** | | **Odds Ratios** | **Std. Err.** | **P-value** | **95%CI** | |  |
| 5-8 years |  |  |  |  | | 1.00 |  |  |  |  | 1.00 |  |  |  | |  |
| 9-10 years |  |  |  |  | | 0.90 | 0.16 | 0.55 | 0.64 | 1.26 | 2.63 | 0.85 | 0.003 | 1.39 | 4.96 |  |
| 11-12 years |  |  |  |  | | 1.29 | 0.26 | 0.22 | 0.86 | 1.92 | 3.70 | 1.56 | 0.002 | 1.62 | 8.45 |  |
| 13-17 years |  |  |  |  | | 1.07 | 0.21 | 0.73 | 0.73 | 1.57 | 2.35 | 0.94 | 0.03 | 1.08 | 5.14 |  |
| Female children |  |  |  |  | |  |  |  |  | | 0.74 | 0.12 | 0.05 | 0.54 | 1.01 |  |
| Caretaker is fishermen or fishmonger |  |  |  |  | |  |  |  |  | | 0.98 | 0.37 | 0.96 | 0.46 | 2.07 |  |
| Caretaker is rice-farmer |  |  |  |  | |  |  |  |  | | 0.61 | 0.19 | 0.12 | 0.33 | 1.14 |  |
| Caretaker is subsistence farmer |  |  |  |  | |  |  |  |  | | 0.66 | 0.15 | 0.07 | 0.42 | 1.04 |  |
| Low level of infection of caretakers (1-99 EPG) | 2.02 | 0.34 | <0.001 | 1.46 | 2.82 | 2.01 | 0.34 | <0.001 | 1.45 | 2.80 | 1.33 | 0.27 | 0.15 | 0.90 | 1.98 |  |
| Moderate level of infection of caretakers (100-399 EPG) | 2.72 | 0.83 | 0.001 | 1.50 | 4.94 | 2.81 | 0.86 | 0.001 | 1.54 | 5.11 | 1.40 | 0.52 | 0.36 | 0.68 | 2.90 |  |
| Heavy level of infection of caretakers (400+ EPG) | 4.39 | 1.60 | <0.001 | 2.15 | 8.97 | 4.42 | 1.62 | <0.001 | 2.16 | 9.02 | 2.67 | 1.11 | 0.02 | 1.18 | 6.01 |  |
| Village with a beach |  |  |  |  | |  |  |  |  | | 3.86 | 0.89 | <0.001 | 2.45 | 6.08 |  |
| Village with a rice paddy |  |  |  |  | |  |  |  |  | | 0.57 | 0.12 | 0.005 | 0.38 | 0.85 |  |
| 3+ roads in the village |  |  |  |  | |  |  |  |  | | 1.21 | 0.36 | 0.51 | 0.68 | 2.17 |  |
| Lake at >0.50km from the village centre |  |  |  |  | |  |  |  |  | | 0.92 | 0.26 | 0.78 | 0.54 | 1.59 |  |
| School at > 1.0km from the village centre |  |  |  |  | |  |  |  |  | | 0.30 | 0.08 | <0.001 | 0.18 | 0.51 |  |
| Age X distance village lake |  |  |  |  | |  |  |  |  | |  |  |  |  | |  |
| 9-10 x >0.50km |  |  |  |  | |  |  |  |  | | 0.26 | 0.11 | <0.001 | 0.12 | 0.57 |  |
| 11-12 x >0.50km |  |  |  |  | |  |  |  |  | | 0.34 | 0.17 | 0.032 | 0.12 | 0.91 |  |
| 13-17 x >0.50km |  |  |  |  | |  |  |  |  | | 0.48 | 0.23 | 0.13 | 0.19 | 1.25 |  |
| List of abbreviations: Std. Err = Standard error, CI = confidence intervals  916 observations in each model  The area under the curve (AUC) value for 5-fold cross validation of the logistic regression model was 0.81. | | | | | | | | | | | | | | | | |
| These models are part of the subgroup analysis of children. Model 1 reports the odds ratios of the logistic regression model for S. mansoni infection risk in children adjusted for infection status of their caretakers only. Model 2 present the odds ratios for the logistic regression model for infection risk in children adjusted for infection status of the caretakers and age of the children. Model 3 presents the odds ratios for the fully adjusted logistic regression model for S. mansoni infection risk in children. | | | | | | | | | | | | | | | | |

**Fig S1:** Individuals involved in fishing by distance from Lake Victoria


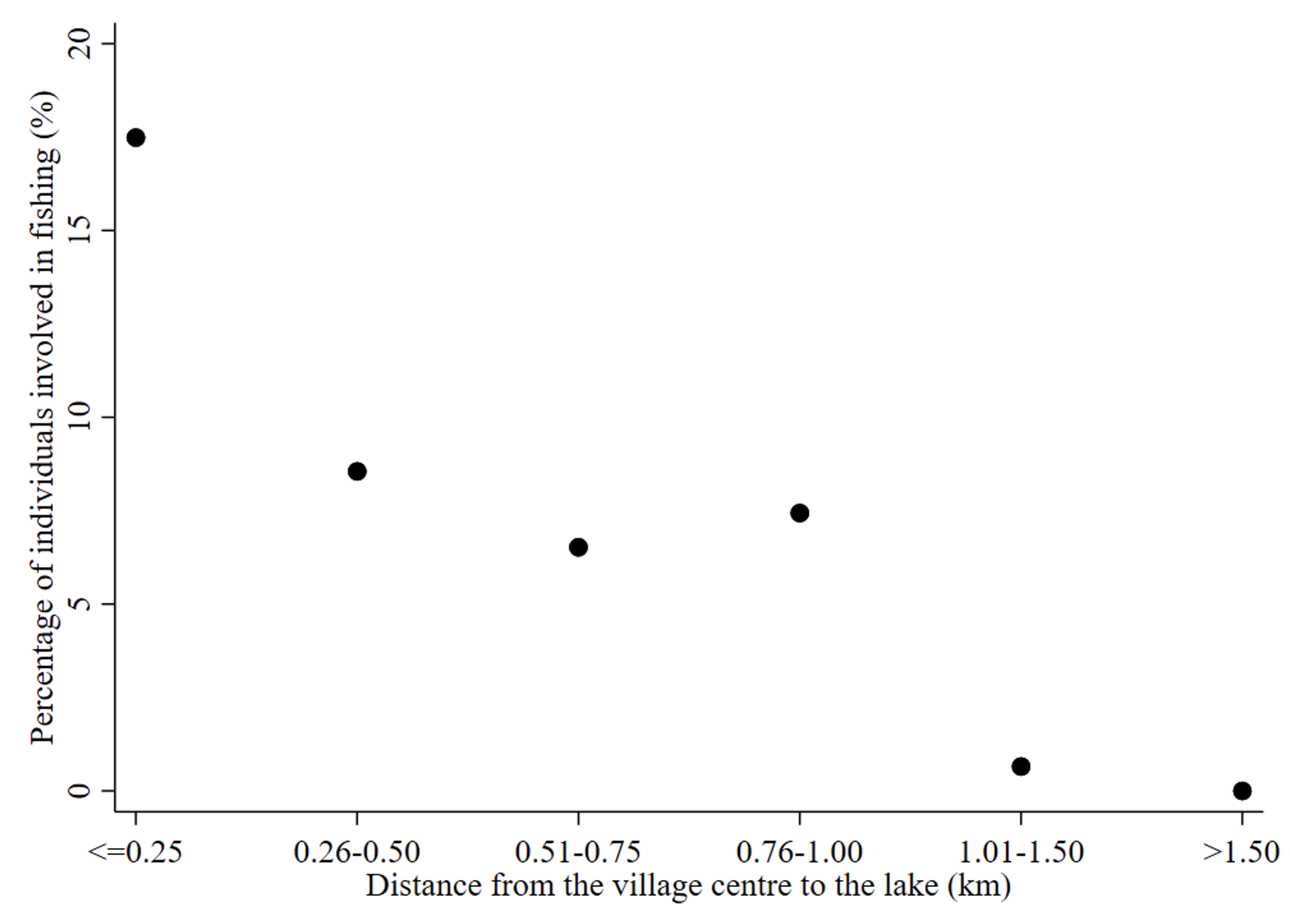


A scatter plot is shown for the percentage of caretakers (adults) involved in fishing by distance from the village centre to the lake. The total number of adults involved in fishing/fishmongering was 17.49% (32/183), 8.55% (13/152), 6.52% (12/184), 7.44% (9/121), 0.65% (1/153), 0% (0/123) for respectively, ≤0.25km, 0.26-.50km, 0.51-0.75km, 0.76-1.00km, 1.01-1.50km, and >1.50km from Lake Victoria. The total number of individuals, including children, for each distance category are reported in Supplementary Table S1.

**References**

1. Tukahebwa EM, Magnussen P, Madsen H, Kabatereine NB, Nuwaha F, Wilson S, et al. A very high infection intensity of *Schistosoma mansoni* in a Ugandan Lake Victoria fishing community is required for association with highly prevalent organ related morbidity. PLoS Negl Trop Dis. 2013;7:e2268.

2. Kabatereine NB, Kemijumbi J, Ouma JH, Kariuki HC, Richter J, Kadzo H, et al. Epidemiology and morbidity of *Schistosoma mansoni* infection in a fishing community along Lake Albert in Uganda. Trans R Soc Trop Med Hyg. 2004;98:711-8.

3. Pinot de Moira A, Fulford AJ, Kabatereine NB, Ouma JH, Booth M, Dunne DW. Analysis of complex patterns of human exposure and immunity to Schistosomiasis mansoni: the influence of age, sex, ethnicity and IgE. PLoS Negl Trop Dis. 2010;4:e820.

4. Exum NG, Kibira SPS, Ssenyonga R, Nobili J, Shannon AK, Ssempebwa JC, et al. The prevalence of schistosomiasis in Uganda: A nationally representative population estimate to inform control programs and water and sanitation interventions. PLoS Negl Trop Dis. 2019;13:e0007617.

5. Woolhouse ME. Patterns in parasite epidemiology: the peak shift. Parasitol Today. 1998;14:428-34.
